# Unravelling the hybrid vigor in domestic equids: the effect of hybridization on bone shape variation and covariation

**DOI:** 10.1186/s12862-019-1520-2

**Published:** 2019-10-15

**Authors:** Pauline Hanot, Anthony Herrel, Claude Guintard, Raphaël Cornette

**Affiliations:** 1 Department of Archaeology, Max Planck Institute for the Science of Human History, Kahlaische Straße 10, D-07745 Jena, Germany; 2 UMR 7179 “ Mécanismes Adaptatifs et Évolution ” (CNRS, MNHN), Muséum national d’Histoire naturelle, Sorbonne Universités, 57 rue Cuvier CP 55, 75005 Paris, France; 3 Unité d’Anatomie Comparée, Ecole Nationale Vétérinaire de l’Agroalimentaire et de l’Alimentation, Nantes Atlantique - ONIRIS, cedex 03, route de Gachet, CS 40706, 44307 Nantes, France; 4 Groupe d’Études Remodelage osseux et bioMatériaux (GEROM), Université d’Angers, Unité INSERM 922, LHEA/IRIS-IBS, CHU d’Angers, 4 rue Larrey, Angers, France; 5 UMR 7205 « Institut de Systématique, Évolution, Biodiversité » (CNRS, MNHN, UPMC, EPHE), Muséum national d’Histoire naturelle, Sorbonne Universités, 45 rue Buffon, 75005 Paris, France

**Keywords:** Appendicular skeleton, Bone morphology, Domestic equids, Hybridization, Three-dimensional geometric morphometrics

## Abstract

**Background:**

Hybridization has been widely practiced in plant and animal breeding as a means to enhance the quality and fitness of the organisms. In domestic equids, this hybrid vigor takes the form of improved physical and physiological characteristics, notably for strength or endurance. Because the offspring of horse and donkey is generally sterile, this widely recognized vigor is expressed in the first generation (F1). However, in the absence of recombination between the two parental genomes, F1 hybrids can be expected to be phenotypically intermediate between their parents which could potentially restrict the possibilities of an increase in overall fitness. In this study, we examine the morphology of the main limb bones of domestic horses, donkeys and their hybrids to investigate the phenotypic impact of hybridization on the locomotor system. We explore bone shape variation and covariation to gain insights into the morphological and functional expressions of the hybrid vigor commonly described in domestic equids.

**Results:**

Our data reveal the occurrence of transgressive effects on several bones in the F1 generation. The patterns of morphological integration further demonstrate that the developmental processes producing covariation are not disrupted by hybridization, contrary to functional ones.

**Conclusions:**

These results suggest that an increase in overall fitness could be related to more flexibility in shape change in hybrids, except for the main forelimb long bones of which the morphology is strongly driven by muscle interactions. More broadly, this study illustrates the interest of investigating not only bone shape variation but also underlying processes, in order to contribute to better understanding how developmental and functional mechanisms are affected by hybridization.

## Background

Although hybridization may lead to the production of less competitive phenotypes [1–3], cases of an increase in fitness and a selective advantage over the parents have been documented in many taxa [4–6]. This phenomenon, generally considered as the result of heterozygosis in hybrids [7, 8], is known as hybrid vigor, or heterosis, and is usually measured by the capacity of hybrids to expand their ecological range and outperform their parent species under natural conditions [9–11]. Heterotic effects can also be artificially targeted in human-mediated hybridizations. This is especially the case in agricultural yield in which hybrids between cultivated and wild forms are created in order to increase crop yield with hybrid plants producing more seeds or fruits than their parents [12, 13]. Similarly, the production of hybrids is also common in animal breeding, as a way to increase the physical, physiological or even cognitive characteristics of the livestock [14, 15]. Among the most familiar examples are the hybrids between domestic equids, mules and hinnies. Mules, the offspring of a male donkey and a female horse (*Equus asinus* x *Equus caballus*), are renowned for the fact that they are taller, faster and more powerful than donkeys as well as for their increased endurance, hardiness and longevity in comparison with horses [16]. These hybrids have also been described as displaying greater cognitive capacities than either parent [17]. Hinnies, the product of mating a male horse with a female donkey (*Equus caballus* x *Equus asinus*) are less common and usually reported to not demonstrate the hybrid vigor generally described for mules [16], mainly because of their smaller stature supposed to be partly related to the mother’s size [18].

Although the hybrid vigor attributed to equid hybrids is widely recognized, especially for mules, its phenotypic expression remains unclear, particularly from an osteological point of view. The fact that mules and hinnies are (with only a few exceptions) sterile [19, 20] probably contributes to the low attention paid to the morphological consequences of hybridization in domestic equids. Indeed, evolutionary studies usually focus on this process in a context of speciation or introgression [21–24]. Moreover, no macroscopic feature specific to hybrid bones has been, for now, identified (in a context in which horse and donkey bones are themselves hard to distinguish) [25], which would suggest the absence of strongly transgressive morphologies related to hybridization in domestic equids.

In a standard polygenic additive model (i.e. one trait controlled by multiple genes with alleles having a similar and additive effect), hybrids are expected to be phenotypically intermediate between their parents [5, 26], especially in the F1 generation, due to the absence of recombination between the two parental genomes [27, 28]. However, non-additive genetic effects can also be involved. They can result in dominance effects (corresponding to the production of hybrid organisms phenotypically closer to one of the parents [29]) or contribute to produce morphologies falling outside the range of variation of both parent species, called transgressive phenotypes [27, 30]. Transgressive effects have already been demonstrated in F1 hybrids [31, 32] with an increased shape disparity and the production of original morphologies prone to play a role in the ability for hybrids to exhibit a wider range of fitness than their parents. The impact of hybridization on morphological traits, coupled with the frequently observed heterotic effect on size, can then induce mechanical or functional changes with respect to parental species which could provide fitness advantages (or disadvantages).

In hybrids from domestic equids, a large part of the widely described hybrid vigor is related to physical performance which is targeted to fit human needs and uses. In that respect, limb bones, even though they are rarely investigated for studying consequences of hybridization, are of particular interest. Indeed, they are the main anatomical elements involved in equid locomotion and their shape can thus be used as a skeletal indicator of functional specificities. Moreover, knowing that performance and functional efficiency of the locomotor system largely results from the mechanical interactions among elements [33, 34], exploring patterns of interactions between bones could provide insights into the functional expression of hybrid vigor. For this reason, we here do not only explore the shape variation but also covariation between bones. The tendency of morphological traits to covary is called morphological integration [35–37] and is produced by various mechanisms such as the sharing of a common function (e.g. modules of within-limb adjacent bones sharing common articulations or muscles) or the sharing of a same developmental origin (i.e. modules of serial homologous bones between fore- and hind limb; Hall, [38]). This incongruence between functional and developmental modules in the appendicular skeletons of tetrapods provides the opportunity to better understand what underlies the shape variation of a bone [39–45]. It is also a way to explore the functional characteristics of an animal but also the degree of conservation of patterns due to developmental processes. This is especially interesting in the study of hybridization knowing that hybridization has been suspected to sometimes disrupt developmental regulation [46, 47]. This issue is moreover particularly relevant in the case of the horse and the donkey, known to show (in comparison with other hybridizing mammals) a high genetic distance [24].

In the present study, we quantify, using 3D geometric morphometrics, the consequences of hybridization on the shape variation of the major limb bones in hybrids from domestic equids. One of the main limitations for characterizing the effect of hybridization in domestic equids is the absence, for these taxa, of available osteological samples including parents and offspring from a same lineage (as well as the near absence of hybrids of known descent which would have, at least, permitted comparisons with the parental breeds used to produce them). In that respect, in order to minimize the inevitable bias related to the sample composition, we tried to characterize at best the morphological variability within each species by including a large diversity of specimens in terms of breed, size, and conformation. We aim to examine the manner in which the “vigor”, defined according to human requirements, is expressed in morphological traits, from first hybrid generation (F1) onwards. To do that, we try to identify potential shape and size differences between hybrids and their parent species and to detect markers of phenotypic transgression. We also investigate the impact of hybridization on phenomena such as allometry (the influence of size on shape, expected to possibly contribute to transgressive effects on shape) and patterns of integration, in order to better understand the underlying causes of morphological variation in hybrids and their parent species.

## Results

The analyses involves the bones of 101 complete or sub-complete skeletons of adult (with fully fused epiphyses) domestic horses of various breeds (*n* = 42), domestic donkeys and wild asses (*n* = 38) and their hybrids (*n* = 21; Table 1, see Additional file 1 for more details). In the absence of significant differences in shape between mules and hinnies [48], they were grouped in following analyses due to the small size of the hybrid sample (13 mules and 8 hinnies).

**Table 1:** List and sample sizes of the breeds included in the analyses

Species	Breed	Sample size
*E. caballus*	Arabian	5
*E. caballus*	Thoroughbred	2
*E. caballus*	Selle Français	3
*E. caballus*	Trotteur français	1
*E. caballus*	Lusitano	1
*E. caballus*	Unknown (riding horse)	1
*E. caballus*	Boulonnais	1
*E. caballus*	Percheron	2
*E. caballus*	Nordiker	2
*E. caballus*	Clydesdale	2
*E. caballus*	Shire	1
*E. caballus*	Unknown (pony)	4
*E. caballus*	Shetland pony	4
*E. caballus*	Icelandic	4
*E. caballus*	Camargue	1
*E. caballus*	Pottok	3
*E. caballus*	Mongol	4
*E. caballus*	Konik	1
*E. asinus*	Egyptian	2
*E. asinus*	Poitou	6
*E. asinus*	Asinara	1
*E. asinus*	Unknown	29
*E. asinus x E. caballus*	Unknown	13
*E. caballus x E. asinus*	Unknown	8

## Shape analyses

Pairwise comparisons show significant shape differences between the three groups in all the bones (MANOVA, *p* < 0.05). The two-way MANOVA performed on the shape data does not indicate an interaction between species-specific and sexual differences in our sample except for the coxal bone. Consequently, coxal shape variation was explored in males and females independently plotting first PCs. Trangression and dominance degrees were also computed to compare with results obtained on the whole dataset but the limited size of our sample does not enable us to perform statistical tests for each sex separately (see Additional file 3).

**Fig. 1:**
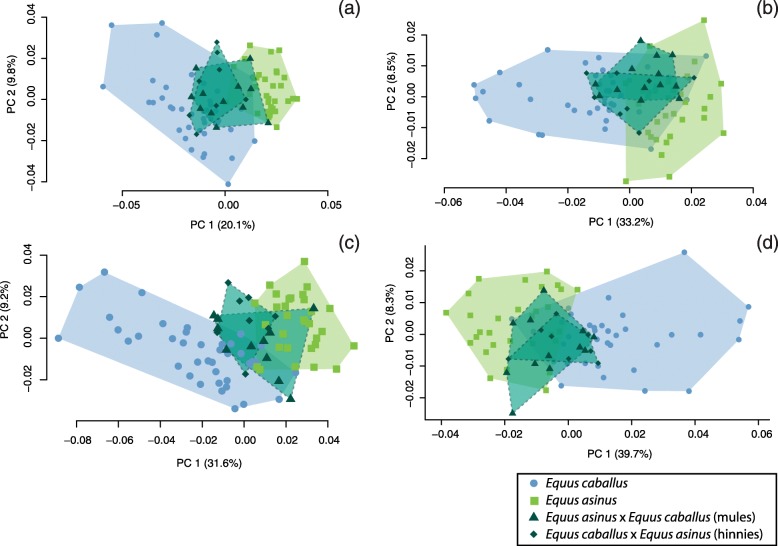
Scatter plot of the two first PCs of the PCA performed on the shape data of the humerus (**a**), radio-ulna (**b**), femur (**c**), tibia (**d**)

The distribution of the specimens along the first axes of the PCA reveals that, for all the bones, most of the hybrid individuals overlap with parental species (Fig. 1; see Additional file 4). Hybrids systematically display an intermediate position along the axis expressing differentiation between parental species (in most cases the PC1, in few instances the PC2) and generally do not exceed the variation of parental groups.

No significant difference in morphological disparity was observed between hybrid and donkey bones except for the middle posterior phalanx in which Procrustes variance is significantly lower in hybrids (Table 2). Concerning the comparison with horses, three bones display a significant difference in morphological disparity corresponding systematically to lower Procrustes variances in hybrids than in horses (metacarpal bone, femur and middle posterior phalanx).

**Table 2:** Estimation of morphological disparity within each group (Procrustes variances), in bold when significantly different from hybrids (*p* < 0.05)

	Scapula	Humerus	Radio-ulna	Metacarpal bone	Proximal anterior phalanx	Middle anterior phalanx	Distal anterior phalanx	Coxal bone
	Variance	Variance	Variance	Variance	Variance	Variance	Variance	Variance
Donkeys	5,23E-03	1,96E-03	9,28E-04	5,97E-04	3,35E-03	1,04E-02	2,10E-02	6,97E-03
Horses	5,39E-03	2,24E-03	1,11E-03	**9,73E-04**	1,87E-02	3,07E-02	3,45E-02	1,44E-02
Hybrids	7,34E-03	1,66E-03	6,90E-04	4,82E-04	2,20E-03	7,30E-03	1,56E-02	5,09E-03
	Femur	Tibia	Talus	Calcaneus	Metatarsal bone	Proximal posterior phalanx	Middle posterior phalanx	Distal posterior phalanx
	Variance	Variance	Variance	Variance	Variance	Variance	Variance	Variance
Donkeys	2,48E-03	1,03E-03	2,17E-02	1,53E-02	4,56E-04	3,89E-03	**1,30E-02**	1,82E-02
Horses	**3,04E-03**	1,23E-03	7,79E-03	6,96E-03	6,50E-04	4,47E-03	**1,18E-02**	1,60E-02
Hybrids	1,62E-03	7,65E-04	6,77E-03	6,77E-03	3,22E-04	2,39E-03	7,48E-03	1,35E-02

## Size and allometry

**Fig. 2:**
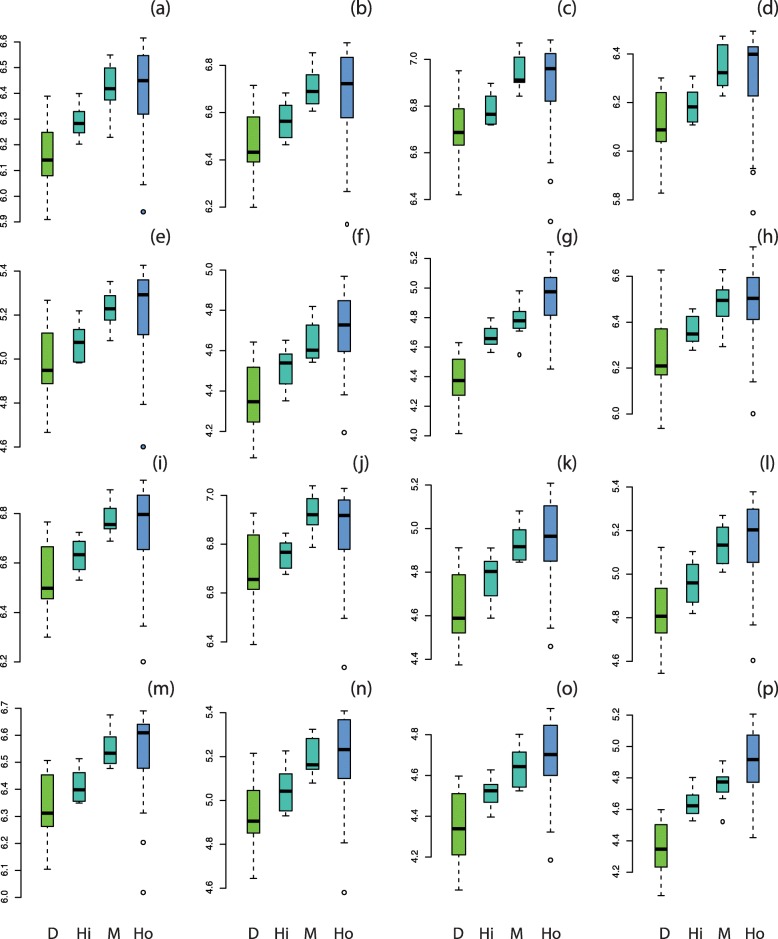
Boxplots of the variation in log-transformed centroid size of the scapula (**a**), humerus (**b**), radio-ulna (**c**), metacarpal bone (**d**), proximal anterior phalanx (**e**), middle anterior phalanx (**f**), distal anterior phalanx (**g**), coxal bone (**h**), femur (**i**), tibia (**j**), talus (**k**), calcaneus (**l**), metatarsal bone (**m**), proximal posterior phalanx (**n**), middle posterior phalanx (**o**), distal posterior phalanx (**p**). Abbreviations: D, donkeys; Hi, hinnies; M, mules; Ho, horses

The results of the ANOVA analyses on size data reveal significant differences between donkeys and horses and between donkeys and hybrids for all the bones (*p* < 0.05), corresponding to smaller bone size in donkeys (Fig. 2). However, bone size is generally not significantly different between horses and hybrids except for distal phalanges (smaller in hybrids than in horses; Fig. 2). The small number of hybrid specimens does not enable us to statistically test potential differences in size between the two hybrid groups but the boxplots suggest that the bone centroid size is higher in mules than in hinnies.

For all the bones, allometry is significant (see Additional file 5). Slope parallelism between groups was supported for half of the bones: metacarpal bone, anterior phalanges, talus, calcaneus and proximal posterior phalanx. However, the percentage of shape variance related to size is low except for distal phalanges and talus.

## Shape transgression and dominance

**Fig. 3:**
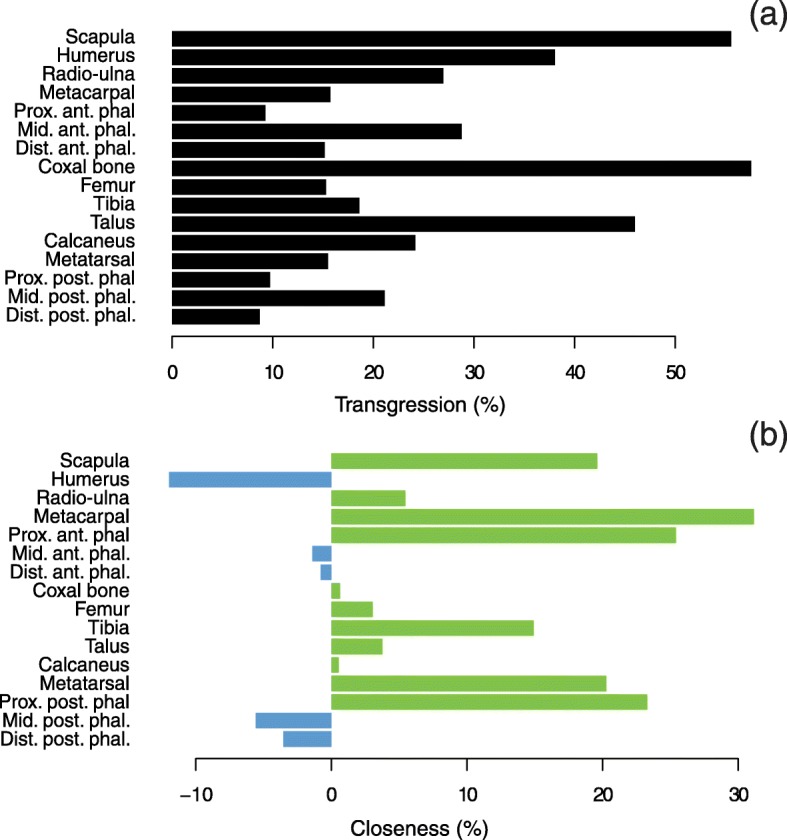
**a** Percentage of transgression of hybrids. **b** Percentage of closeness to parent species with positive values indicating greater closeness to donkey (in green) and negative ones to horse (in blue)

The girdles (scapula and coxal bone) reveal the highest degrees of transgression (> 50%) followed by the talus (46%, Fig. 3a). This transgression observed for the coxal bone is reinforced in the analyses computed on males and females separately (see Additional file 3). The forelimb proximal long bones (humerus and radio-ulna, respectively 38 and 27%) are more transgressive than the hind limb ones (femur and tibia, respectively 15 and 19%). Concerning the distal bones (metapodials and phalanges), they generally display the lowest degrees of transgression (< 20%) except for the middle phalanges which appear as more transgressive in both limbs.

The results concerning the degree of dominance of the parental strains to hybrid morphology reveal a greater closeness to donkeys (Fig. 3b), on most of the bones, with the highest values (> 20%) found on the proximal autopodial bones (metapodial bones and proximal phalanges). On the contrary, a greater closeness to horses is observed for the humerus, and albeit to a lesser extent, middle and distal phalanges.

## Shape integration patterns

**Fig. 4:**
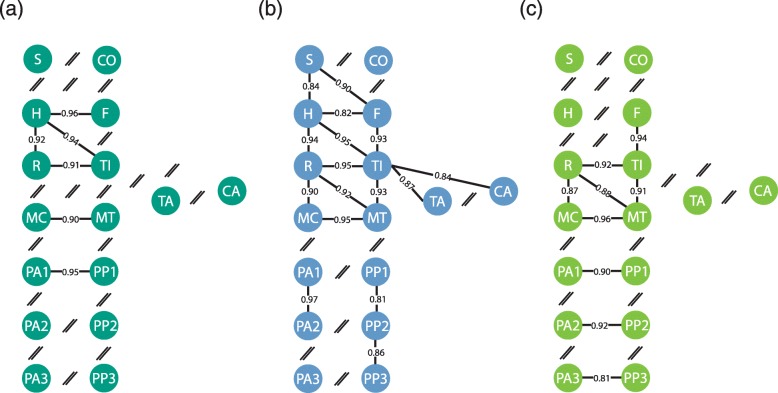
Graphical models of the rPLS coefficients obtained on the appendicular bones of hybrids (**a**), horses (**b**) and donkeys (**c**). The line thickness is proportional to the coefficient values (the boldest lines corresponding to the strongest intensity of covariation). The absence significant of covariation (non-significant PLS result) is represented by a double slash ‘//’. Abbreviations: S, scapula; H, humerus; R, radio-ulna; MC, metacarpal bone; PA1, proximal anterior phalanx; PA2, middle anterior phalanx; PA3, distal anterior phalanx; C, coxal bone; F, femur; T, tibia; MT, metatarsal bone; TA, talus; CA, calcaneus; PP1, proximal posterior phalanx; PP2, middle posterior phalanx; PP3, distal posterior phalanx

The covariation between the serial homologous bones is significant (*p* < 0.05) for most of the pairs in hybrids (Fig. 4). The absence of significant covariation between girdles (scapula and coxal bone) is also observed in both parent species, and that between middle and distal phalanges is shared with horses.

Concerning the intra-limb adjacent bones, only the covariation between the humerus and radio-ulna is significant in hybrids. In that respect, they differ from both parent species by the absence of significant covariation between the femur and tibia, and between the zeugopods and metapodials (radio-ulna/metacarpal bone, tibia/metatarsal bone).

Similarly, among the functional equivalent bones, only the covariation between the humerus and tibia is significant. Whereas the absence of significant covariation between the scapula and femur is also noticed in donkeys, the absence of significant covariation between the radio-ulna and metatarsal bone contrasts with both parent species.

When significant, the rPLS values in hybrids are globally high (rPLS ≥0.90) suggesting a strong degree of covariation between bones. However, intensity of integration in hybrids is globally lower than in horses, with significant differences in the z-scores for most of the pairs (see Additional file 6).

**Fig. 5:**
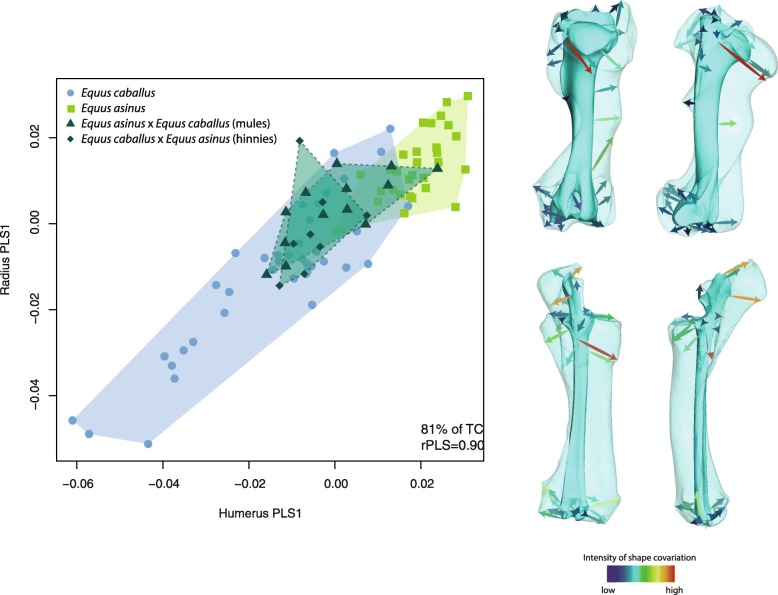
Scatter plot of the first PLS axis describing shape covariation between the humerus and radio-ulna, with visualizations of the associated shape changes (in transparent: extreme negative; in opaque: extreme positive). The color of the arrows corresponds to the intensity of shape deformation along the PLS axis (red: high intensity; blue: low intensity). TC: total covariation; r_PLS_: PLS correlation coefficient

The first axis of covariation between the humerus and radio-ulna explain 90% of the total covariance. The distribution of the specimens along this axis follows a similar pattern than in the PCA with hybrids displaying an intermediate position between their parent species (Fig. 5). The morphological changes related to this axis are largely related to bone robusticity and curvature of the diaphysis (with horses displaying more robust and curved bones than donkeys). More precisely, some anatomical areas mainly contribute to the covariation: the proximal part of the caudal surface of the humerus (corresponding to the origin of the brachialis and triceps brachii muscles); the radial tuberosity on the medial side of the radius (corresponding to the insertion of the brachialis muscle) and the olecranon tuberosity on the ulna (corresponding to the insertion of the triceps brachii muscle).

## Discussion

### Pattern of shape differentiation between the hybrids and their parent species

Hybridization has been previously reported as able to produce an increased shape variance from the first generation hybrids onwards [31]. In the absence of recombination between the parental genomes, this phenomenon has been related to decreased canalization in hybrids (i.e. the buffering of developmental processes against genetic and environmental variation in order to keep the phenotype constant). In our case, the absence of significant increased shape variance within the hybrid group in comparison with their parent species does not allow us to suggest that canalization is affected by hybridization in domestic equids. However, the impossibility to directly compare hybrids with their actual parents (or at least with a sub-sample comprised of the parental breeds used to produce the hybrids in our dataset) makes it impossible to discuss the potential impact of hybridization on shape variance in our sample. Further studies comparing shape variance between equid hybrids and their actual parents would be of interest to be able to shed light on the potential effect of hybridization on shape variance and canalization.

Although hardly discernible on the visualization of two first PCs, our results reveal transgression in hybrid shape. Indeed, the fact that shape differences between hybrids and both their parent species is significant for all the bones suggests that hybridization in domestic equids is able to produce a globally original morphology. The assessment of transgression in hybrid shape reveals that they do not display a strictly intermediate position between the parental strains with a percentage of transgression of over 40% of the between-parents distance on some bones. This confirms the idea that transgression may occur as soon as the first generation in hybrids [31, 32], in spite of the absence of genetic recombination expected to result in an intermediate morphology [26, 49]. In our dataset, we observe dominance effects with a greater closeness of hybrids to donkeys for most of the bones. This refutes the simple additive genetic model of strictly intermediate shape between parental strains for hybrids.

### Size and allometry

A common effect of hybridization is the phenomenon of heterosis (or hybrid vigor) which corresponds to the improved quality of some biological and physiological characteristics of a hybrid organism, over those of its parents, generally providing a fitness advantage. Heterotic effects were noticed in hybrids from numerous taxa and may notably be expressed by an increase in size [29, 31, 47, 50, 51].

In our case, the fact that hybrid bones are significantly larger than those of donkeys and not significantly different from those of horses suggests that they exceed the size intermediacy between parent species, expected under the additive model. However, reliably characterizing such a heterotic effect on hybrid bone size is difficult for the same reasons than mentioned above: it would require a comparison of hybrids from our sample to parental groups constituted of similar breeds. Our current sample is indeed very disparate due to the great diversity in size of the modern parental species, especially horses (from Shetland ponies to draft breeds in the present study). A larger sample of hybrids specimens would also be of interest in order to be able to analyze mules separately from hinnies. Indeed, hinnies contribute to lower the mean size in the hybrid group whereas their smaller size is probably more related to the influence of physiological mechanisms (especially the mother size) rather than genetic ones [18].

Although we are unable to confidently confirm heterotic effect in size in our domestic equid sample, this phenomenon should not be neglected as a potential factor for transgression in shape. This is why allometry was examined in order to assess its possible contribution to the transgressive effect on shape, in cases of significant differences in bone size between the hybrids and their parent species (all the bones in donkeys, distal phalanges in horses). However, the low percentage of the shape variance related to size in most of the bones, coupled with the absence of homogeneity in the allometric slopes among species, suggest that transgression in hybrid shape is not driven by size differences (and hence potential heterotic effects on size).

The only exceptions are the anterior distal phalanges and the talus, in which a high percentage of shape variance is related to size, with hybrids and parent species sharing a common allometric trend. This consistency in the intergroup allometry suggests that their differentiation in shape could be partly due to allometric effects and size differences, probably revealing their role in guiding evolutionary changes in the distal parts.

### Consequences of hybridization on development

In order to determine the factors that possibly contribute to explain these shape differences, we investigated morphological integration between the bones. In hybrids, covariation between serial-homologous bones is significant in most cases, contrasting with intra-limb adjacent bones. This reveals that the integration pattern in hybrid limbs is largely shaped by development and suggests that hybridization, although resulting from the combination of two different genomes, does not disrupt the developmental processes associated to the relationships between fore- and hind limbs. It should also be mentioned that some pairs of serial-homologous bones significantly covary in hybrids, even when this is not the case in both parent species. This confirms the strong impact of development in shaping covariation in hybrids, but also its underlying variation. Indeed, the significant covariation between the humerus and femur inherited from horses possibly contributes to driving the singular closeness of the humerus to that of horses. Similarly, the significant covariation between the proximal phalanges observed in hybrids, also only noticeable in donkeys, may echo the especially strong closeness of these two elements to donkeys.

The absence of significant covariation between middle and between distal phalanges is shared with horses, but also with various other tetrapods [39, 40] and could be explained by the proximo-distal increase in variability along the limb [39]. Similarly, the absence of significant covariation between girdles is also noticeable in parent species [52] and possibly results from the singular developmental pathway of the scapula [53, 54]. On another note, this relative morphological independence of the girdles from the rest of the skeleton in horses and donkeys echoes the high degree of transgression and the relative high shape variance noticed for these bones in hybrids. Indeed, the high degree of modularity of the parental scapula and the coxal bone could have contributed to facilitate shape variation in hybrids by increasing evolvability [55]. A similar finding applies to the talus which quite lowly covaries with adjacent elements in horses and donkeys, and is among the most transgressive bones in hybrids.

### Consequence of hybridization on function

The absence of significant covariation between most of the intra-limb adjacent bones and functionally equivalent bones in hybrids reveals that functional factors do not contribute much to integration in the appendicular skeleton. This contrasts with horses and donkeys and suggests that hybridization may be accompanied by the relaxation of some processes producing phenotypic covariation between limb bones. Assuming that modularity would contribute to facilitate shape changes, loosening of covariation imputed to function could allow for a wider range of functional adaptations in hybrids, generally expressed by transgression and increased morphological variability [11, 31].

The only significant signal of integration between intra-limb adjacent bones concerns the humerus and radio-ulna, showing that the functional relationships between these two bones continues to generate shape covariation. More precisely, the interactions related to muscle action seem to underlie this covariation. Indeed, the PLS analysis reveals that the main areas of covariation correspond to the origin and insertion areas of the major muscles linking the humerus and radio-ulna, including the brachialis and triceps brachii muscles. The changes in diaphysis curvature could also be partly related to functional interactions, especially to the bending strains imposed by locomotion and muscular forces [56, 57]. The singular occurrence of significant covariation between the main forelimb bones involved in the muscular system probably suggests their functional importance in hybrid locomotion. In most tetrapods, the forelimb is mainly involved in support and braking [58] whereas the hind limb is optimized for generating propulsion [59]. In that respect, our result could appear as surprising considering that the intensity of morphological integration is higher in the hind limb of several taxa due to its major role in locomotion [40]. This was also observed on domestic equids [52] and could contribute to explain the lower degree of transgression noticed in the hind limb compared to the forelimb in hybrids. Indeed, the stronger intensity of integration between the femur and tibia in parent species could have participated to constraint transgression in hybrids.

The singular covariation between the humerus and radio-ulna in hybrids reminds the pattern of dominance noticed for the humerus, which contrasts from most of the bones by displaying greater closeness to the parental horse morphology. Knowing that functional interactions have been shown as contributing to bone shape in this anatomical area, the greater closeness to horses could be partly related to function, especially muscle characteristics. This is consistent with the fact that force and power are definitively the main features selected in horses and likely inherited by hybrids in their production [60]. Moreover, the hypothesis that special functional mechanisms underlie and drive the shape variation of the humerus is supported by the singular significant covariation with its functional equivalent, even absent in donkeys.

## Conclusions

This study sought to assess the impact of hybridization on the morphology of the limb bones in domestic equids. Our results reveal that, for several bones, hybrids do not strictly display an intermediate position between their parent species, horses and donkeys, unlike what might be expected for first generation hybrids. Indeed, transgressive effects were brought to light, contributing to the global shape differentiation between hybrids and parent species. Our results show that the developmental mechanisms producing covariation are not disrupted by hybridization, contrary to functional ones. This would suggest that, surprisingly, the widely recognized vigor of these hybrids does not rest on a strongly integrated system which would drive bone shape variation as a result of functional interactions. Indeed, modularity might, on the contrary, contribute to facilitate shape changes, potentially as a way to increase overall fitness. The significant morphological covariation observed between the humerus and radio-ulna is an exception. It appears to be mostly related to muscle interactions and probably reveals the particular importance of this anatomical area in support and braking.

Additional studies comparing hybrids with their actual parents are needed to demonstrate the potential occurrence of phenomena generally associated to hybridization and prone to play a role in producing hybrid vigor including heterotic effects in size. Measuring the amount of fluctuating asymmetry as a marker of developmental (in) stability could also be a way to detect hybrid vigor as it is assumed to be correlated to overall fitness [51, 61].

More generally, this study contributes to show the importance of investigating the impact of hybridization on bone shape variation, but also on the processes which underlie it to gain insight into how developmental and functional mechanisms are affected. Our study also illustrates the interest of examining the appendicular skeleton which is prone to document the effect of hybridization on the locomotor system. This aspect, which is only rarely considered, can contribute to enrich our vision about the functional expression of hybrid vigor.

## Methods

### Acquisition of data

A 3D geometric morphometric approach was applied to the 16 main limb bones of the equid skeleton (scapula, humerus, radio-ulna, metacarpal bone, coxal bone, femur, tibia, calcaneus, talus, metatarsal bone, proximal, middle, and distal anterior and posterior phalanges). The 3D coordinates of anatomical landmarks were recorded according to the protocol of Hanot et al. [48] using a Microscribe 3D digitizer. Due to the fragmented nature of some bones, some landmarks were removed in the analyses (see Additional file 2).

For each bone, a generalized Procrustes Analysis (GPA) was performed on the landmark data in order to remove non-shape variation due to differences in position, scale, and orientation of the configurations [62].

### Shape analyses

A Principal Component Analysis (PCA) was performed on the procrustes residuals of each bone to reduce the dimensionality of the multivariate datasets [63–66]. The first Principal Components (PCs) were then plotted in order to observe the distribution of the data in shape space. The PCAs were performed using the R package “Rmorph” [67, 68].

Shape differences between species (horses, donkeys and hybrids) were tested for each bone using Multivariate Analyses of Variance (MANOVA) with pairwise comparisons and Bonferroni corrections for multiple testing. They were performed on the Principal Components (PCs) explaining 90% of the total variance using the the “RVAideMemoire” library [69].

A two-way MANOVA was performed on the shape data to test the potential interaction between species differentiation and sexual dimorphism (on bones displaying a significant difference in shape between males and females). However, due to the small number of geldings in the sample, their potential impact on the shape variation was not taken into account.

The Procrustes variance of each bone was computed to assess the morphological disparity within each group using the “geomorph” library [70]. It was completed by pairwise comparisons among groups with Bonferroni corrections for multiple testing.

### Size and allometry

Size differences between species were tested for each bone using Analyses of Variance (ANOVA) associated to pairwise post-hoc T-tests, using the “lsr” library [71], with Bonferroni corrections for multiple testing. Variations in size according to species were figured using box plots of the log10-transformed centroid sizes.

Allometry was assessed using multivariate regressions of shape variables on the log10-transformed centroid sizes. The homogeneity of allometric slopes among groups was then tested using the “geomorph” library [70].

### Shape transgression and dominance

Closeness and transgression were quantified according to the method proposed by Renaud et al. (2012). First, for each bone, the group mean of each species was computed and the Euclidean distances between them measured using the “Morpho” library [72].

The degree of transgression was assessed by computing the percentage of the summed distance between the hybrids and each parent species on the distance between parent species themselves: (dDHy+dHoHy-dDHo)*100/ dDHo (with “d” for Euclidean distance, “Hy” for hybrids, “D” for donkeys and “Ho” for horses). Therefore, the higher this percentage, the stronger the transgression, considering that the absence of transgression (i.e. the strictly intermediate position of the hybrids between the parent species) should correspond to the equal distance between parent species (dDHo) and between hybrids and each parent species (dDHy+dHoHy).

The degree of dominance was assessed by comparing the average distance between the hybrids and their parent species to the distance between the hybrids and donkeys, as a percentage of the average distance between the hybrids and their parent species: ((dDHy+dCHy)/2-dDHy)*100/((dDHy+dCHy)/2). Hence, positive values indicate that hybrids display a greater closeness to donkeys and negative values to horses.

### Shape integration patterns

The shape covariation between bones was explored for all the within-limb adjacent and serially homologous bones. We also investigated the covariation between the functionally equivalent bones from fore- and hind limb [44, 52, 73], defined according to the reorganization of the skeleton in therian mammals [74, 75].

In order to assess the intensity of shape co-variation between bones, Partial Least Squares coefficients (rPLS) were computed on the Procrustes shape variables. The Two blocks-PLS analysis extracts the eigenvectors and eigenvalues from blocks of variables and examines the covariance between them [76, 77]. A significant covariation is obtained when the observed rPLS is higher than those of a random distribution of values from permuted blocks. High values for rPLS correspond to strong morphological integration and vice versa. Bonferroni corrections were conducted to adjust the *p*-values from each set of tests including a same bone.

In addition, the z-scores (effect size) of rPLS values were computed in order to enable their comparison between datasets [78]. This test estimates the standard deviate of rPLS values obtained from different datasets displaying different expected values under the null hypothesis of no integration (due to variations in the number of variables and sample size). The difference in the effect-sizes was then assessed using two-sample tests with Bonferroni corrections for multiple testing. These analyses were computed using the “geomorph” library [70].

In some cases, in order to better investigate the shape covariation, the first PLS axes were plotted using the “Rmorph” library [67] and the associated shape changes were visualized. Visualizations of the extreme shapes associated with the PLS axes were produced using a 3D photogrammetric model (from the bones of the modern specimen: CV9 - ONIRIS-Nantes AC, with photographs taken using a Canon EOS 700D and 3D reconstructions computed on the software Agisoft PhotoScan;© 2014 Agisoft LLC, 27 Gzhatskaya st., St. Petersburg, Russia). The landmark coordinates of the 3D model were obtained from the “IDAV Landmark Editor” [79] and the shape deformation along the axes were finally visualized thanks to a Thin Plate Spline (TPS) deformation of the consensus surface, using the “Morpho” library [72].

For all the analyses previously described, test results were considered as significant when p-values (*p*) were below 0.05.

## Supplementary information

**Additional file 1.** List of the specimens included in the analyses (table)

**Additional file 2.** List of the anatomical landmarks from the protocol of Hanot et al. 2017a which are not retained in the analyses due to the poor preservation of some bones (table).

**Additional file 3.** Scatter plot of the two first PCs of the PCA performed on the shape data of the coxal bone of females and males (figure); Percentage of transgression and closeness to parent species of hybrids (table).

**Additional file 4.** Scatter plot of the two first PCs of the PCA performed on the shape data (figure).

**Additional file 5 ***P*-values and coefficient of determination of the multivariate regressions of shape variables on the log10-transformed centroid sizes (table).

**Additional file 6.** Graphical models of z-scores (effect size) of rPLS values obtained on the appendicular bones of hybrids, horses and donkeys (figure).

## Data Availability

The datasets used and analyzed during the current study are available from the corresponding author on request.

## Abbreviations

ANOVA: Analysis of Variance
C: Coxal bone
CA: Calcaneus
D: Donkey
d: Euclidean distance
F: Femur
F1: First generation
GPA: Generalized Procrustes Analysis
H: Humerus
Hi: Hinny
Ho: Horse
Hy: Hybrid
M: Mule
MANOVA: Multivariate Analysis of Variance
MC: Metacarpal bone
MT: Metatarsal bone
*p*: *P*-value
PA: Anterior phalanx
PC: Principal Component
PCA: Principal Component Analysis
PLS: Partial Least Squares
PP: Posterior phalanx
R: Radio-ulna
rPLS: PLS correlation coefficient
S: Scapula
T: Tibia
TA: Talus
TC: Total Covariation
TPS: Thin Plate Spline
