# Supplementary material for: Unravelling the hybrid vigor in domestic equids: the effect of hybridization on bone shape variation and covariation
Source: BMC Evol Biol. 2019 Oct 15;19:188. doi: 10.1186/s12862-019-1520-2 (PMC6794909; doi:10.1186/s12862-019-1520-2)
Supplement: Supplementary file 1 — Additional file 1. List of the specimens included in the analyses (table) [file 12862_2019_1520_MOESM1_ESM.pdf]

Electronic Supplementary Material 1:

List of the specimens

Collection	Inventory no	Species	Breed	Sex
MNHN-Paris (AC)	MNHN-ZM-AC-1927-235	*E. caballus*	Arabian	M
IRSNB-Brussels	IRSNB-3.975	*E. caballus*	Arabian	M
ZSM-Munich	1965-205	*E. caballus*	Arabian	F
MLU/ZNS/H-Halle	E arb 3	*E. caballus*	Arabian	-
MLU/ZNS/H-Halle	E arb 2	*E. caballus*	Arabian ?	F
MNHN-Paris (AC)	MNHN-ZM-AC-1914-337	*E. caballus*	Thoroughbred	M
ONIRIS-Nantes (AC)	CV9	*E. caballus*	Thoroughbred	F
ONIRIS-Nantes (AC)	CV1	*E. caballus*	Selle Français	F
ONIRIS-Nantes (AC)	CV3	*E. caballus*	Selle Français	F
ONIRIS-Nantes (AC)	CV4	*E. caballus*	Selle Français	F
ONIRIS-Nantes (AC)	CV2	*E. caballus*	Trotteur Français	F
ONIRIS-Nantes (AC)	CV6	*E. caballus*	Lusitano	F
ONIRIS-Nantes (AC)	CV7	*E. caballus*	Unknown (riding horse)	F
MNHN-Paris (AC)	MNHN-ZM-AC-1891-107	*E. caballus*	Boulonnais	M
MNHN-Paris (AC)	MNHN-ZM-AC-1890-1178	*E. caballus*	Percheron	F
MSN-Angers	MHNAn-2010-440	*E. caballus*	Percheron	M
NHM-Wien (AZ)	1767	*E. caballus*	Noriker (Pinzgauer)	M
NHM-Wien (AZ)	A538	*E. caballus*	Noriker (Pinzgauer)	M
MLU/ZNS/H-Halle	E cldd 1	*E. caballus*	Clydesdale	M
MLU/ZNS/H-Halle	E cldd 4	*E. caballus*	Clydesdale	F
MLU/ZNS/H-Halle	E shr 1	*E. caballus*	Shire	M
MNHN-Paris (AC)	MNHN-ZM-AC-1937-51	*E. caballus*	Unknown (Pony)	F
MNHN-Paris (AC)	MNHN-ZM-AC-1937-59	*E. caballus*	Unknown (Pony)	M
ONIRIS-Nantes (AC)	CV5	*E. caballus*	Unknown (Pony)	F
ONIRIS-Nantes (AC)	CV8	*E. caballus*	Unknown (Pony)	M
MNHN-Paris (AC)	MNHN-ZM-AC-1945-27	*E. caballus*	Shetland pony	F
IRSNB-Brussels	IRSNB-13.097	*E. caballus*	Shetland pony	F
SAPM-Munich	SAPM-MA-01141	*E. caballus*	Shetland pony	F
ZSM-Munich	1963-29	*E. caballus*	Shetland pony	F
MNHN-Paris (AC)	MNHN-ZM-AC-1873-385	*E. caballus*	Icelandic	F
MNHN-Paris (AC)	MNHN-ZM-AC-1903-135	*E. caballus*	Icelandic	-
MNHN-Paris (AC)	MNHN-ZM-AC-1975-98	*E. caballus*	Icelandic	M
ZSM-Munich	1961-29	*E. caballus*	Icelandic	F
IRSNB-Brussels	IRSNB-13.071	*E. caballus*	Camargue	F
IRSNB-Brussels	IRSNB-14.209	*E. caballus*	Pottok	-
IRSNB-Brussels	IRSNB-16.958	*E. caballus*	Pottok	F
IRSNB-Brussels	IRSNB-16.959	*E. caballus*	Pottok	F
MLU/ZNS/H-Halle	E mgl 1	*E. caballus*	Mongol	F
MLU/ZNS/H-Halle	E mgl 2	*E. caballus*	Mongol	F

MLU/ZNS/H-Halle	E mgl 3	*E. caballus*	Mongol	F
MLU/ZNS/H-Halle	E mgl 4	*E. caballus*	Mongol	F
CRAVO-Compiègne	CRAVO Konik	*E. caballus*	Konik	F
MNHN-Paris (AC)	MNHN-ZM-AC-1875-028	*E. asinus*	Egyptian	F
MNHN-Paris (AC)	MNHN-ZM-AC-1893-634	*E. asinus*	Egyptian	M
MNHN-Paris (AC)	MNHN-ZM-AC-1982-128	*E. asinus*	Poitou	F
MNHN-Paris (AC)	MNHN-ZM-AC-2012-63	*E. asinus*	Poitou	F
MNHN-Paris (AC)	MNHN-ZM-AC-A551	*E. asinus*	-	-
MNHN-Paris (AC)	MNHN-ZM-AC-1933-397	*E. asinus*	-	M
MNHN-Paris (AC)	MNHN-ZM-AC-1901-69	*E. asinus*	-	F
MNHN-Paris (AC)	MNHN-ZM-AC-2009-588	*E. asinus*	-	-
MNHN-Paris (UMR 7209)	without no	*E. asinus*	-	-
IRSNB-Brussels	IRSNB-7.501	*E. asinus*	Poitou	G
IRSNB-Brussels	IRSNB-7.502	*E. asinus*	-	F
IRSNB-Brussels	IRSNB-12.970	*E. asinus*	-	F
IRSNB-Brussels	IRSNB-13.075	*E. asinus*	-	G
IRSNB-Brussels	IRSNB-13.076	*E. asinus*	-	F
IRSNB-Brussels	IRSNB-93.35.M1	*E. asinus*	-	-
IRSNB-Brussels	IRSNB-93.35.M3	*E. asinus*	-	-
IRSNB-Brussels	IRSNB-93.35.M4	*E. asinus*	-	-
SAPM-Munich	SAPM-MA-01097	*E. asinus*	-	M
SAPM-Munich	SAPM-MA-02358 (a)	*E. asinus*	-	M
SAPM-Munich	SAPM-MA-02358 (b)	*E. asinus*	-	-
SAPM-Munich	SAPM-MA-02360	*E. asinus*	-	M
SAPM-Munich	SAPM-MA-02362	*E. asinus*	-	F
SAPM-Munich	SAPM-MA-02364	*E. asinus*	Poitou	M
SAPM-Munich	SAPM-MA-02369	*E. asinus*	Poitou	F
SAPM-Munich	SAPM-MA-02373	*E. asinus*	-	F
ZSM-Munich	1952-9	*E. asinus*	-	F
ZSM-Munich	1954-110	*E. asinus*	Asinara	F
ZSM-Munich	1961-7	*E. asinus*	-	M
ZSM-Munich	1961-144	*E. asinus*	-	M
ZSM-Munich	1963-134	*E. asinus*	-	M
MLU/ZNS/H-Halle	Ea pt 1	*E. asinus*	Poitou	F
MLU/ZNS/H-Halle	Ea 11	*E. asinus*	-	F
MLU/ZNS/H-Halle	Ea 12	*E. asinus*	-	F
MLU/ZNS/H-Halle	Ea 13	*E. asinus*	-	F
MLU/ZNS/H-Halle	Ea 15	*E. asinus*	-	F
NHM-Wien (AZ)	1326	*E. asinus*	-	-
NHM-Wien (AZ)	A170	*E. asinus*	-	F
NHM-Wien (AZ)	E4	*E. asinus*	-	M
MNHN-Paris (AC)	MNHN-ZM-AC-A543	*E. asinus x E. caballus*	-	M
IRSNB-Brussels	IRSNB-13.156	*E. asinus x E. caballus*	-	M
NHM-Wien (AZ)	E1058	*E. asinus x E. caballus*	-	F
ZSM-Munich	1970-5	*E. asinus x E. caballus*	-	M
ZSM-Munich	1970-6	*E. asinus x E. caballus*	-	F
ZSM-Munich	1972-337	*E. asinus x E. caballus*	-	F
ZSM-Munich	1972-338	*E. asinus x E. caballus*	-	M

SAPM-Munich	SAPM-MA-01024	*E. asinus x E. caballus*	-	F
SAPM-Munich	SAPM-MA-01025	*E. asinus x E. caballus*	-	G
SAPM-Munich	SAPM-MA-01026	*E. asinus x E. caballus*	-	G
SAPM-Munich	SAPM-MA-01027	*E. asinus x E. caballus*	-	G
MHJK-Halle	mlt1	*E. asinus x E. caballus*	-	F
MHJK-Halle	mlt3	*E. asinus x E. caballus*	-	-
MHJK-Halle	mls1	*E. caballus x E. asinus*	-	F
MHJK-Halle	mls2	*E. caballus x E. asinus*	-	F
MHJK-Halle	mls3	*E. caballus x E. asinus*	-	M
MHJK-Halle	mls4	*E. caballus x E. asinus*	-	F
MHJK-Halle	mls5	*E. caballus x E. asinus*	-	F
MHJK-Halle	mls6	*E. caballus x E. asinus*	-	M
MHJK-Halle	mls7	*E. caballus x E. asinus*	-	F
MHJK-Halle	mls10	*E. caballus x E. asinus*	-	G

Table: List of the specimens included in the analyses.
Abbreviations for institutions: MNHN-Paris (AC), Muséum national d'Histoire naturelle (Comparative Anatomy) – Paris/France; MNHN-Paris (UMR 7209),: Muséum national d'Histoire naturelle (UMR 7209 « Archéozoologie, Archéobotanique ») – Paris/France; MSN-Angers, Muséum des Sciences naturelles de la ville d'Angers – Angers/France; ONIRIS-Nantes (AC), Ecole Nationale Vétérinaire, Agroalimentaire et de l'Alimentation Nantes-Atlantique (Comparative Anatomy) – Nantes/France; IRSNB-Brussels, Institut royal des Sciences naturelles de Belgique – Brussels/Belgium; ZSM-Munich, Zoologische Staatssammlung Muenchen – Munchen/Germany; NHM-Wien (AZ), Naturhistorisches museum Wien (Archeological Zoological Collection) – Vienna/Austria; SAPM-Munich, Staatssammlung für Anthropologie und Paläoanatomie München – Munchen/Germany ; MLU/ZNS/H-Halle -Halle, Zentralmagazin Naturwissenschaftlicher Sammlungen der Martín-Luther-Unversität Halle-Wittenberg (Museum für Haustierkunde « Julius Kühn ») - Halle (Saale)/Germany. Abbreviations for sex: F, female ; M, male ; G, gelding.
