# Supplementary material for: Unravelling the hybrid vigor in domestic equids: the effect of hybridization on bone shape variation and covariation
Source: BMC Evol Biol. 2019 Oct 15;19:188. doi: 10.1186/s12862-019-1520-2 (PMC6794909; doi:10.1186/s12862-019-1520-2)
Supplement: Supplementary file 2 — Additional file 2. List of the anatomical landmarks from the protocol of Hanot et al. 2017a which are not retained in the analyses due to the poor preservation of some bones (table). [file 12862_2019_1520_MOESM2_ESM.pdf]

Electronic Supplementary Material 2:

Anatomical landmarks

Bone	Removed anatomical landmarks from the protocol of Hanot et al. 2017a
Scapula	3
Humerus	18, 40, 43
Radius-ulna	6, 18, 42
Metacarpal bone	10, 12, 32
Proximal anterior phalanx	8, 9, 18
Middle anterior phalanx	-
Distal anterior phalanx	-
Coxal bone	23,24
Femur	20,31,32,38
Tibia	18, 19
Talus	13, 20, 25
Calcaneus	14,17
Metatarsal bone	-
Proximal posterior phalanx	18
Middle posterior phalanx	-
Distal posterior phalanx	-

Table: List of the anatomical landmarks from the protocol of Hanot et al. 2017a which are not retained in the analyses due to the poor preservation of some bones.
