# Supplementary material for: Unravelling the hybrid vigor in domestic equids: the effect of hybridization on bone shape variation and covariation
Source: BMC Evol Biol. 2019 Oct 15;19:188. doi: 10.1186/s12862-019-1520-2 (PMC6794909; doi:10.1186/s12862-019-1520-2)
Supplement: Supplementary file 3 — Additional file 3. Scatter plot of the two first PCs of the PCA performed on the shape data of the coxal bone of females and males (figure); Percentage of transgression and closeness to parent species of hybrids (table). [file 12862_2019_1520_MOESM3_ESM.pdf]

Electronic Supplementary Material 3:

Shape variation in the coxal bone

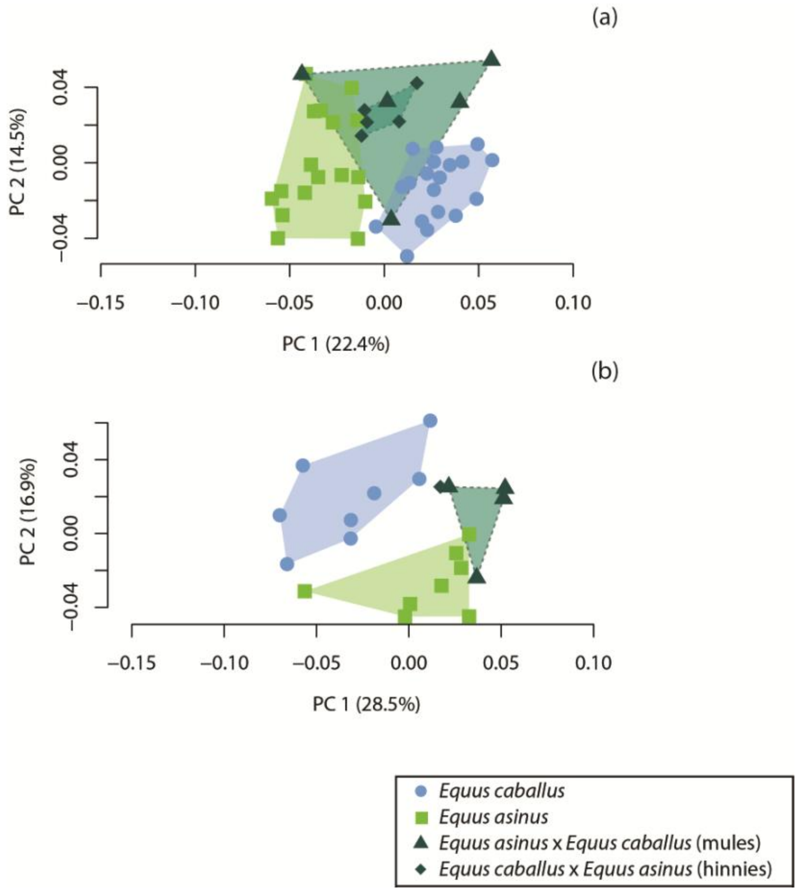

Figure: Scatter plot of the two first PCs of the PCA performed on the shape data of the coxal bone of females (a) and males (b).

	Transgression (%)	Closeness (%)
Females	68.4	-2.7
Males	113.6	12.2

Table: Percentage of transgression and closeness to parent species of hybrids, with positive values indicating greater closeness to donkey and negative ones to horse.
