# Supplementary material for: Unravelling the hybrid vigor in domestic equids: the effect of hybridization on bone shape variation and covariation
Source: BMC Evol Biol. 2019 Oct 15;19:188. doi: 10.1186/s12862-019-1520-2 (PMC6794909; doi:10.1186/s12862-019-1520-2)
Supplement: Supplementary file 4 — Additional file 4. Scatter plot of the two first PCs of the PCA performed on the shape data (figure). [file 12862_2019_1520_MOESM4_ESM.pdf]

Electronic Supplementary Material 4:

Principal Component Analyses plots

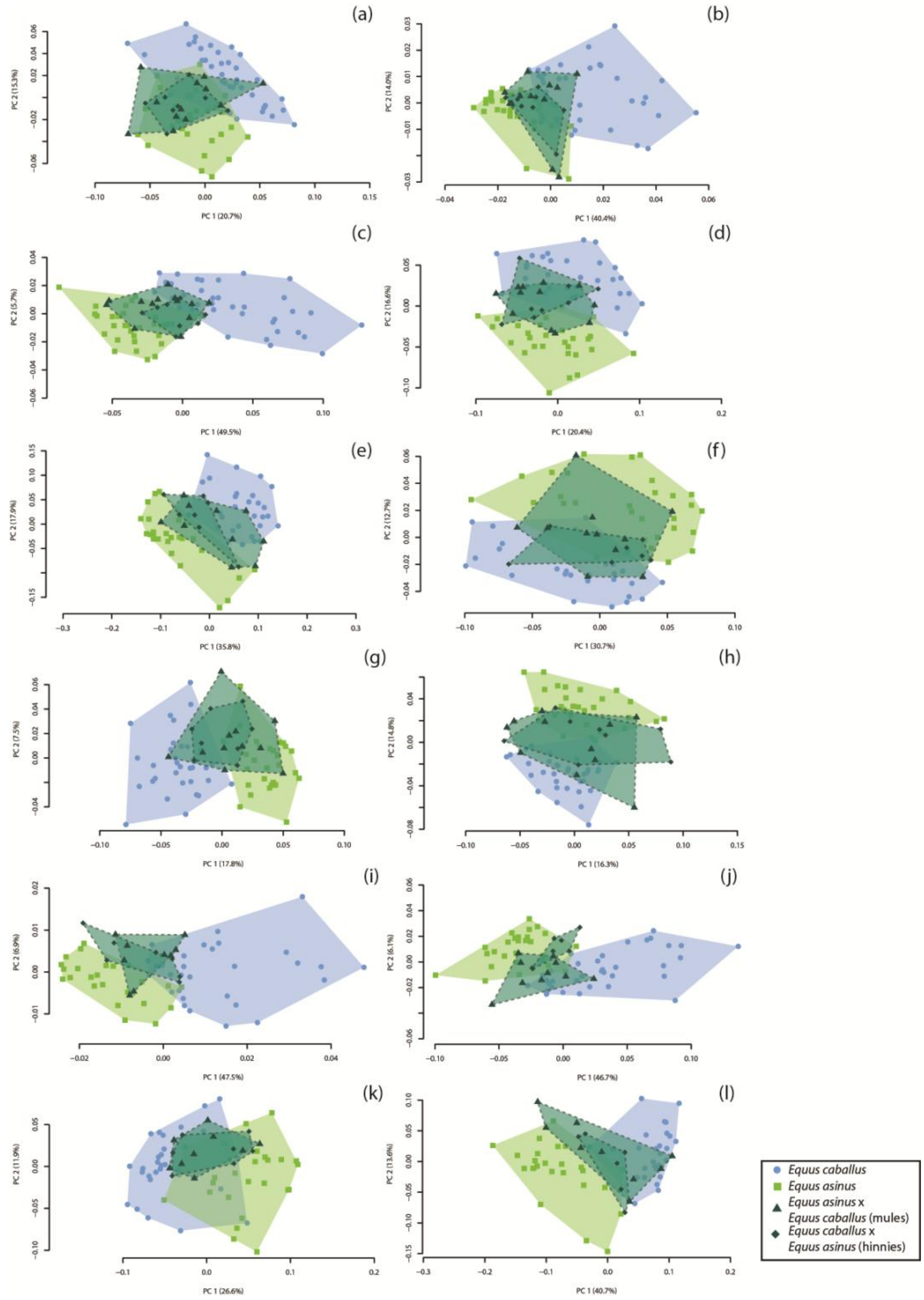

Figure: Scatter plot of the two first PCs of the PCA performed on the shape data of the scapula (a), metacarpal bone (b), proximal anterior phalanx (c), middle anterior phalanx (d), distal anterior phalanx (e), coxal bone (f), talus (g), calcaneus (h), metatarsal bone (i), proximal posterior phalanx (j), middle posterior phalanx (k), distal posterior phalanx (l).
