# Supplementary material for: Unravelling the hybrid vigor in domestic equids: the effect of hybridization on bone shape variation and covariation
Source: BMC Evol Biol. 2019 Oct 15;19:188. doi: 10.1186/s12862-019-1520-2 (PMC6794909; doi:10.1186/s12862-019-1520-2)
Supplement: Supplementary file 5 — Additional file 5 P-values and coefficient of determination of the multivariate regressions of shape variables on the log10-transformed centroid sizes (table). [file 12862_2019_1520_MOESM5_ESM.pdf]

Electronic Supplementary Material 5:

Allometry

	PC1		PC2		90%	
	p	R^2	p	R^2	p	R^2
Scapula	0.33	-	<0.05	0.21	<0.05	0.56
Humerus	<0.05	0.25	0.18	-	<0.05	0.75
Radio-ulna	<0.05	0.19	0.70	-	<0.05	0.65
Metacarpal bone	<0.05	0.17	<0.05	0.08	<0.05	0.50
Proximal anterior phalanx	<0.05	0.06	<0.05	0.16	<0.05	0.51
Middle anterior phalanx	0.33	-	<0.05	0.47	<0.05	0.70
Distal anterior phalanx	<0.05	0.49	<0.05	0.33	<0.05	0.88
Coxal bone	<0.05	0.10	<0.05	0.13	<0.05	0.68
Femur	<0.05	0.40	0.06	-	<0.05	0.70
Tibia	<0.05	0.18	<0.05	0.14	<0.05	0.74
Talus	<0.05	0.14	0.28	-	<0.05	0.73
Calcaneus	<0.05	0.06	<0.05	0.20	<0.05	0.58
Metatarsal bone	<0.05	0.35	0.31	-	<0.05	0.60
Proximal posterior phalanx	0.78	-	<0.05	0.05	<0.05	0.36
Middle posterior phalanx	<0.05	0.18	<0.05	0.24	<0.05	0.72
Distal posterior phalanx	<0.05	0.56	<0.05	0.22	<0.05	0.90

Table: P-values (p) and coefficient of determination (R^2) of the multivariate regressions of shape variables on the log10-transformed centroid sizes.
