# Supplementary material for: Unravelling the hybrid vigor in domestic equids: the effect of hybridization on bone shape variation and covariation
Source: BMC Evol Biol. 2019 Oct 15;19:188. doi: 10.1186/s12862-019-1520-2 (PMC6794909; doi:10.1186/s12862-019-1520-2)
Supplement: Supplementary file 6 — Additional file 6. Graphical models of z-scores (effect size) of rPLS values obtained on the appendicular bones of hybrids, horses and donkeys (figure). [file 12862_2019_1520_MOESM6_ESM.pdf]

Electronic Supplementary Material 6:

Morphological integration (effect size)

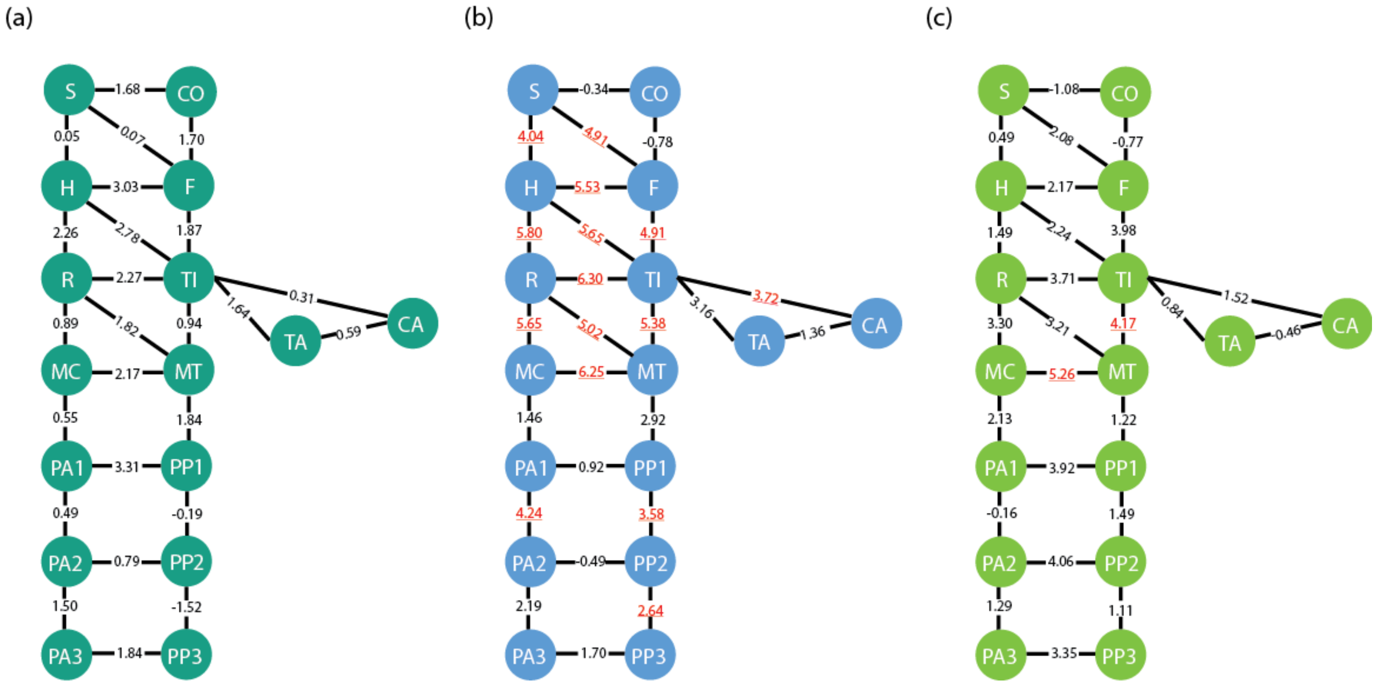

Figure: Graphical models of z-scores (effect size) of rPLS values obtained on the appendicular bones of hybrids (a), horses (b) and donkeys (c). Z-scores significantly different between hybrids and parents are underlined in the parents' pattern. Abbreviations: S, scapula; H, humerus; R, radio-ulna; MC, metacarpal bone; PA1, proximal anterior phalanx; PA2, middle anterior phalanx; PA3, distal anterior phalanx; C, coxal bone; F, femur; T, tibia; MT, metatarsal bone; TA, talus; CA, calcaneus; PP1, proximal posterior phalanx; PP2, middle posterior phalanx; PP3, distal posterior phalanx.
